# The accumulation of deficits approach to describe frailty

**DOI:** 10.1371/journal.pone.0223449

**Published:** 2019-10-15

**Authors:** Raskit Lachmann, Marta Stelmach-Mardas, Manuela M. Bergmann, Wolfgang Bernigau, Daniela Weber, Tobias Pischon, Heiner Boeing

**Affiliations:** 1 Department of Epidemiology, German Institute of Human Nutrition, Potsdam-Rehbruecke, Germany; 2 Department of Biophysics, Poznan University of Medical Sciences, Poznan, Poland; 3 Department of Molecular Toxicology, German Institute of Human Nutrition, Potsdam-Rehbruecke, Germany; 4 Molecular Epidemiology Research Group, Max-Delbrueck Center for Molecular Medicine in the Helmholtz Association (MDC), Berlin-Buch, Germany; Universita degli Studi di Napoli Federico II, ITALY

## Abstract

The advancing age of the participants of the European Prospective Investigation into Cancer and Nutrition (EPIC)—Potsdam study was the incentive to investigate frailty as a major parameter of ageing. The aim of this study was to develop a multidimensional tool to measure frailty in an ageing, free-living study population. The “accumulation of deficits approach” was used to develop a frailty index (FI) to characterize a sub-sample (N = 815) of the EPIC-Potsdam (EPIC-P) study population regarding the aging phenomenon. The EPIC-P frailty index (EPIC-P-FI) included 32 variables from the following domains: health, physical ability, psychosocial and physiological aspects. P–values were calculated for the linear trend between sociodemographic and life style variables and the EPIC-P-FI was calculated using regression analysis adjusted for age. The relationship between the EPIC-P-FI and age was investigated using fractional polynomials. Some characteristics such as age, education, time spent watching TV, cycling and a biomarker of inflammation (C-reactive protein) were associated with frailty in men and women. Interestingly, living alone, having no partner and smoking status were only associated with frailty in men, and alcohol use and physical fitness (VO2max) only in women. The generated, multidimensional FI, adapted to the EPIC-P study, showed that this cohort is a valuable source for further exploration of factors that promote healthy ageing.

## Introduction

Frailty is a concept of impairment in an elderly population and is used to describe the variability in the ageing process between individuals. Studies showed that frailty is associated with falls, malnutrition, hospitalization, institutionalization, disability and death [1–4]. The existence of frailty in an individual could impair an active and satisfying life in advanced age and is becoming increasingly common in an ageing population.

Although it is widely accepted that frailty exists, searching for an operational common definition or measurement tool of frailty is still an ongoing process [5–7]. Frailty definitions are based on two approaches: the phenotype and the accumulation of deficits approach. The phenotype approach uses the biological syndrome model of frailty, measuring weight loss, fatigue, exhaustion, weakness, low physical activity, slowness, and mobility impairment. Currently, one of the most widely used measures of physical frailty is the frailty phenotype index from Fried et al. [8] which based on a yes/no scoring system of 5 variables and assumes frailty if a person has 3 or more points. In contrast to this approach of measuring frailty, the accumulation of deficit approach uses the burden model of frailty, including symptoms, diseases, conditions and disability [9]. This approach was described by, among others, Rockwood and Mitnitski [10] and includes at least 30 deficits from different domains. These domains should have some relations to health [11]. It is still unclear if disabilities and comorbidities themselves should be a domain to define frailty or if they already constitute the adverse health outcomes.

Although the use of the deficit approach is more challenging due to the higher number of variables needed, it was argued that, compared to Fried’s frailty phenotype scale, a frailty deficit index (FI) is a more sensitive predictor for adverse health outcomes due to its finer graded scale and its multidimensionality [12]. This fine grading of the phenomenon of frailty by the deficit approach could be an advantage in prospective cohort studies due to the need of a profound understanding of the outcome “frailty”. This need is not as urgent in the clinical setting in which the Fried phenotype scale of frailty is often used to characterize patients and their treatment needs.

The European Prospective Investigation into Cancer and Nutrition (EPIC) cohort study could benefit from a tool to define frailty in a comparable manner to other studies for further research regarding the aging phenomenon. With advanced aging of this cohort, studying physiological and lifestyle related aspects of frailty, in connection with ageing itself, seems possible due to the increasing numbers of persons potentially affected by frailty [13, 14].

Therefore, the accumulation of deficits approach of measuring frailty was used in this study to provide a multidimensional tool for the investigation of healthy ageing and to describe frailty in the EPIC-Potsdam cohort.

## Materials and methods

### Study population

#### Baseline EPIC study population

EPIC-Potsdam is one out of 23 study centers, which recruited approximately 520,000 participants in 10 European countries in 1992–1998. The EPIC study population represents the general population but was not chosen to provide representative samples. Thus, the recruitment in Potsdam was determined by practical and logistic considerations in order to obtain high participation and long-term follow-up from the study participants. The study procedures and the study population have been described previously [13, 14]. Briefly, the target population was the general population, men aged 40–64 years and women aged 35–64 years living in Potsdam and surrounding municipalities [15]. In the study center, an informed consent form had to be signed and standardized comprehensive interview and physical examinations including the collection of a blood sample had to be completed in order for the person to be accepted as a participant. At the end of the recruitment period from 1994 to 1998, 27,548 subjects had passed successfully through the examination center in Potsdam [15, 16].

#### Re-examined sub-sample of the original study population

**Fig 1:**
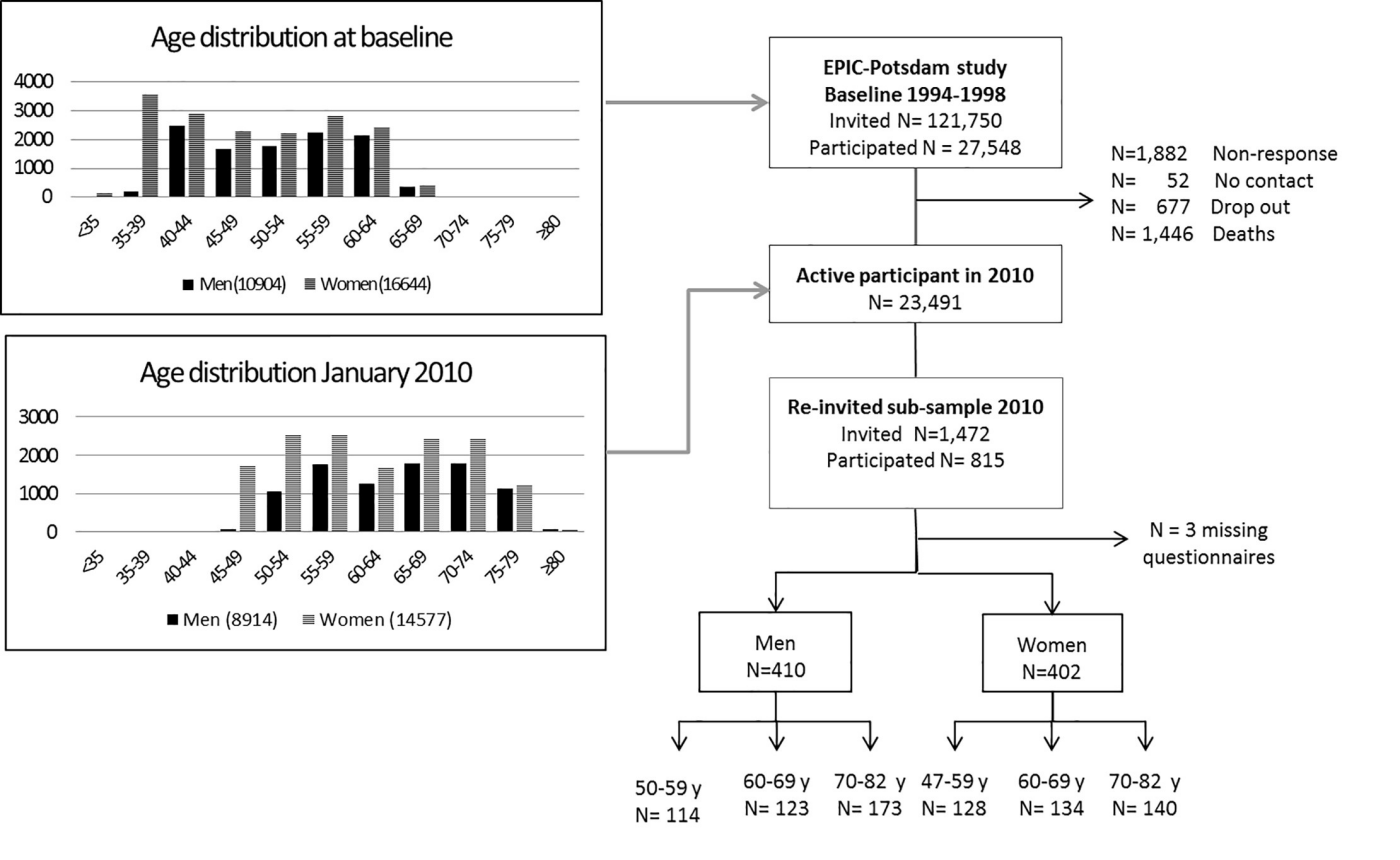
Flow chart of the EPIC-Potsdam participants at baseline and in the re-invited sub-sample 2010 and age distribution. Bar graphs show the number of participants in each 5-year category (a) at baseline 1998 and (b) in 2010 for the entire cohort for men (black) and women (grey).

In 2010, a randomly selected sample stratified by gender and age at the time of recruitment was re-invited for a more detailed assessment of exposure variables. Sub-study participants were recruited randomly from the still available study population according to a rectangular sampling scheme, designed to include approximately 300 men and 300 women with equal representation of each of the three 10-year categories for age at baseline (35–44 years, 45–54 years and 55–64 years). 1472 active participants were invited for re-examination, with 815 (55.2%) completing the examination. All participants underwent the proof of exclusion criteria such as severe disease (i.e. heart attack or cancer 6 months prior to the appointment) and gave written informed consent prior to their examination. Participants completed extensive questionnaires, were anthropometrically re-examined and were equipped with a combined heart rate and movement sensor that objectively measured physical activity (PA) continuously over a 7-day period. Three participants did not answer one or more of the questionnaires and were excluded from the analysis. Thus, data from 812 participants (410 men, 402 women) were analyzed (Fig 1). The sub-study was approved by the ethics committee of the State Medical Association of Brandenburg (reference number S 9/2002 ma-ne and 2110/ne).

### Data collection

Information on socio-demographic factors was available in the database from data collection at recruitment. Data on health, physical ability, psychosocial and physiological aspects were obtained by self-administered questionnaires and physical examinations at the time of the visit of the examination center. Anthropometric variables, including body weight, waist circumference (WC), height and blood pressure (BP) were obtained following standard operating protocols. Body mass index (BMI) was calculated as weight in kilogram (kg) divided by squared height in meters and waist-height-ratio (WHTR) as WC in centimeters divided by height in centimeters. Body fat mass was measured using Bioelectrical Impedance analysis. Hand-grip strength was measured using the Jamar hand dynamometer (Lafayette Instrument Company, USA). The assessment of cardiorespiratory fitness and physical activity has been described in detail previously [17]. In brief, cardiorespiratory fitness was assessed by an 8-min step test (200-mm step; Reebok, Lancaster, UK). Physical activity level (PAL) was calculated from the combined heart rate and movement monitor Actiheart (Cambridge Nurotechnology, Cambridge, UK) which was worn for 8 days by the participants.

### Development of the EPIC-P-FI

The EPIC-P-FI was based on the FI framework developed by Rockwood and Mitnitski [10, 18]. According to the framework, the FI should contain at least 30 deficits, which can be: symptoms, signs, limitations and diseases. The selected deficits should be associated with health status, do not saturate too early, have more than 1% prevalence, and should cover a range of health problems and disabilities [11]. To start with, 39 variables of the EPIC-Potsdam data set reflecting deficits from the four domains of health, physical ability, psychosocial and physiological aspects were considered as potential predictors of frailty for the EPIC-P-FI. Variables were dichotomized (1 = presence of deficit; 0 = absence of deficit) or were assigned to three levels (0, 0.5 or 1 point) to reflect differences in severity. The cut-off points were set, whenever possible, according to the literature. Overweight and normal weight were coded as 0; obesity and underweight were given a point [19, 20]. Furthermore, presence of deficit was defined as: WHTR of more than 0.6 [21], the highest quintile of body fat percentage (adjusted for gender), malnutrition (energy intake under 21 kcal per day per kg bodyweight) [22], PAL under 1.4, (very inactive people) [23], losing more than 5% bodyweight in two years without intending it (unwanted weight loss) [8] and using more than five medications (polypharmacy) [24]. The cut-off for hand-grip-strength was set according to BMI and gender as performed by Fried et al. [8].

Thirty-nine potential variables were analyzed for multicollinearity to exclude variables that were not adding information to the FI. Seven variables were excluded by this procedure leaving 32 variables for the final FI. The EPIC-P-FI was calculated by adding all present deficits divided by the total number of deficits available for each participant to produce an EPIC-P-FI between 0 and 1. The individual domains were calculated in the same way: 12 deficits in health, eight deficits in physical ability, seven deficits in psychosocial aspects and five deficits in physiological aspects. Additionally, in a sensitivity analysis, a reduced FI was calculated with 23 deficits (excluding chronic diseases) and compared with the complete EPIC-P-FI. Prevalent diseases (tumor, diabetes, myocardial infarction, stroke, Transient Ischemic Attack, heart failure, angina pectoris, hypertension, osteoporosis) were included. This originates from the fact that it is still unclear if comorbidities are an essential part of frailty or an adverse health outcome of frailty.

### Statistical analysis

All analyses were carried out independently in men and women [3]. Sensitivity analyses for participants with missing values were performed to see if findings are sensitive to the inclusion or exclusion of these participants from the analysis. Complete data for all EPIC-P-FI deficits were available for 62% (n = 506) of all participants, 25% (n = 200) had one missing value, 10% (n = 79) had two missing values and the remaining 3% (n = 27) had three to five missing values. Since many participants had missing data for grip strength (male: n = 40, female: n = 51) and PAL (male: n = 62, female: n = 67), a Mann-Whitney U test was conducted to analyze if these participants differ from participants with available data for hand-grip strength and PAL respectively.

Multiple imputation procedures were applied for all variables included in the EPIC-P-FI. The SAS procedure PROC MI was used to create 10 imputed data sets with a maximum number of iterations set at 200 with all variables used to construct the EPIC-P-FI. The correlation between the original EPIC-P-FI and the imputed EPIC-P-FI was very high (R^2^ = 0.99, P<0.001), and thus we preferred to use the original EPIC-P-FI for analysis.

Descriptive statistics were used to describe demographic characteristics of the study population. Means were age-adjusted to account for different age distributions. The P -values for trend with increasing EPIC-P-FI values were calculated using regression analysis adjusted for age (for all variables except for the variable age). Differences between groups were calculated with chi-square test for categorical data, Kruskal-Wallis test for continuous data, Fisher’s exact test when expected cell counts were smaller than five. Participants were grouped into the categories low, medium and high EPIC-P-FI values based on gender-specific EPIC-P-FI tertiles. The relationship between the EPIC-P-FI values and age was investigated using fractional polynomials (SAS macro mfp8). P<0.05 was used for establishing statistical significance. Statistical analyses were done using SAS Software, version 9.4.

## Results

### Characteristics of the EPIC-P-FI

Thirty-two variables from four domains (twelve variables in health, seven variables in psychosocial aspects, eight variables in physical ability and five variables in physiological aspects) were included to calculate the EPIC-P-FI. The prevalence of individual EPIC-P-FI variables s presented in the supplemental material (S1 and S2 Tables).

The distribution of the EPIC-P-FI score was skewed to the right, in both men and women from the EPIC-Potsdam study. The EPIC-P-FI had an upper 99% limit of 0.52 for men and women with a range from 0–0.60 (mean = 0.16, median = 0.14) in men and 0–0.70 (mean = 0.18, median = 0.16) in women.

**Fig 2:**
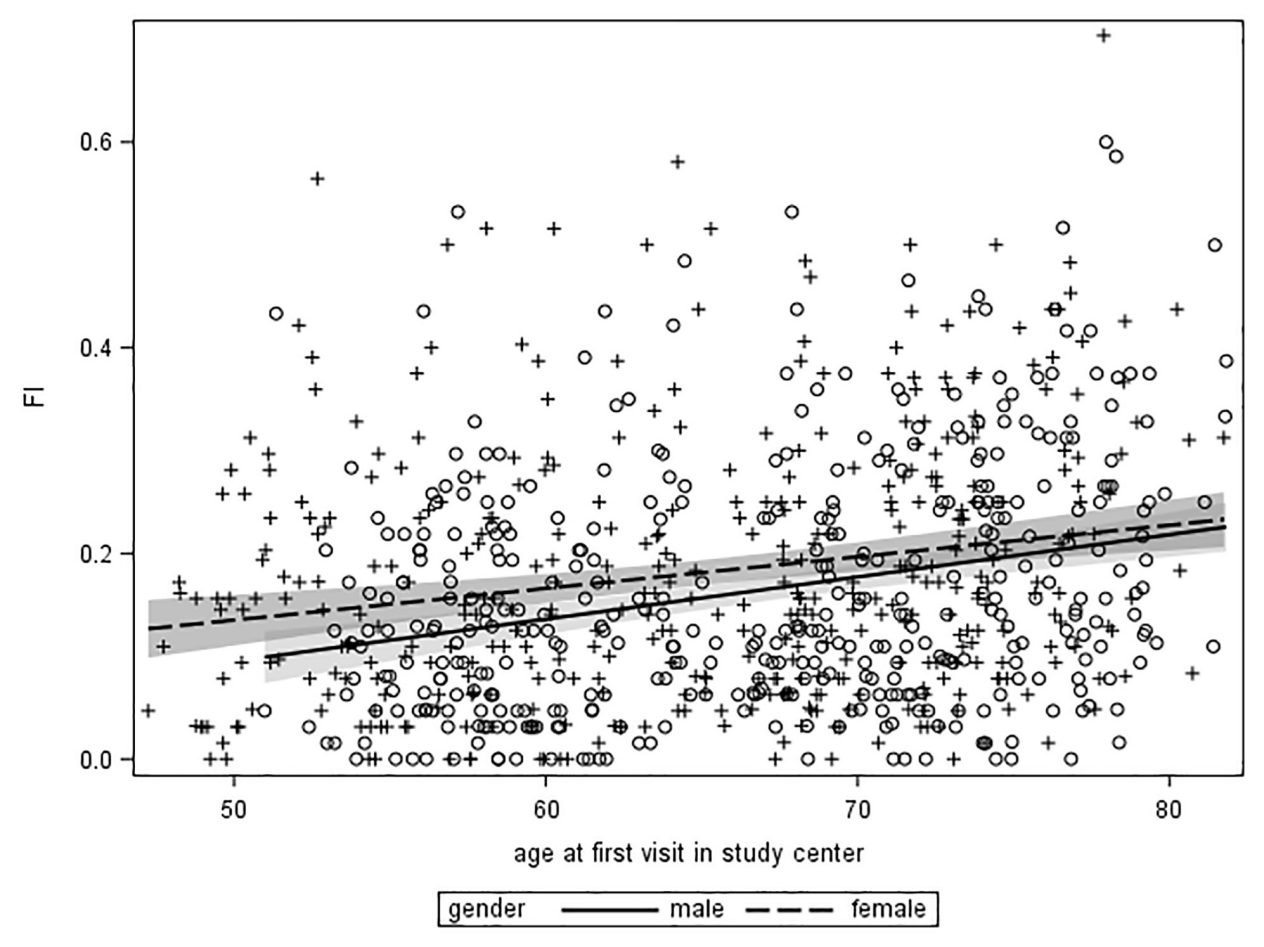
The distribution of the EPIC-P-FI score in relation to age in years in men and women. Dots for individual data points and prediction lines are shown in the scatter plot, the shaded areas denote the 95% CI for the prediction for men (light) and women (dark).

The mean EPIC-P-FI scores were slightly lower in men in all age groups compared to women, and increased across age groups in men (<60 years: FI = 0.13; 60–70 years: FI = 0.15 and >70 years: FI = 0.19, P-trend < .0001) and in women (<60 years: FI = 0.15; 60–70 years: FI = 0.17 and >70 years: FI = 0.22, P-trend < .0001). The EPIC-P-FI correlated linearly with age in men (R2 = 0.07, P<0.001) and women (R2 = 0.04, P<0.001, Fig 2) and increased, on average per year of age, by 0.004 (95% CI: 0.003, 0.006) in men and by 0.003 (95% CI: 0.002, 0.004) in women.

### Correlation of individual domains to the overall EPIC-P-FI

Variables from the four domains of health, physical ability, psychosocial and physiological aspects were combined to calculate the total EPIC-P-FI. In men and women, the domain physical ability showed the highest correlation with the EPIC-P-FI score (ρ = 0.81, P<0.001 and ρ = 0.86, P<0.001 respectively, Table 1).

**Table 1:** Correlation coefficient rho (ρ) between the total EPIC-P-FI score, the four domains and age.

**Men**					
** **	**EPIC-P-FI**	**Psychosocial aspects**	**Health**	**Physical ability**	**Physiological aspects**
**Psychosocial aspects**	0.60^a^	1			
**Health**	0.76^a^	0.23^a^	1		
**Physical ability**	0.81^a^	0.48^a^	0.52^a^	1	
**Physiological aspects**	0.51^a^	0.05	0.23^a^	0.12^a^	1
**Age**	0.27^a^	0.05	0.36^a^	0.30^a^	-0.04
**Women**					
** **	**EPIC-P-FI**	**Psychosocial aspects**	**Health**	**Physical ability**	**Physiological aspects**
**Psychosocial aspects**	0.69^a^	1			
**Health**	0.70^a^	0.29^a^	1		
**Physical ability**	0.86^a^	0.54^a^	0.54^a^	1	
**Physiological aspects**	0.52^a^	0.08	0.19^a^	0.23^a^	1
**Age**	0.21^a^	-0.02	0.30^a^	0.18^a^	0.12^a^

^a^ *P*-value < 0.05

**Fig 3:**
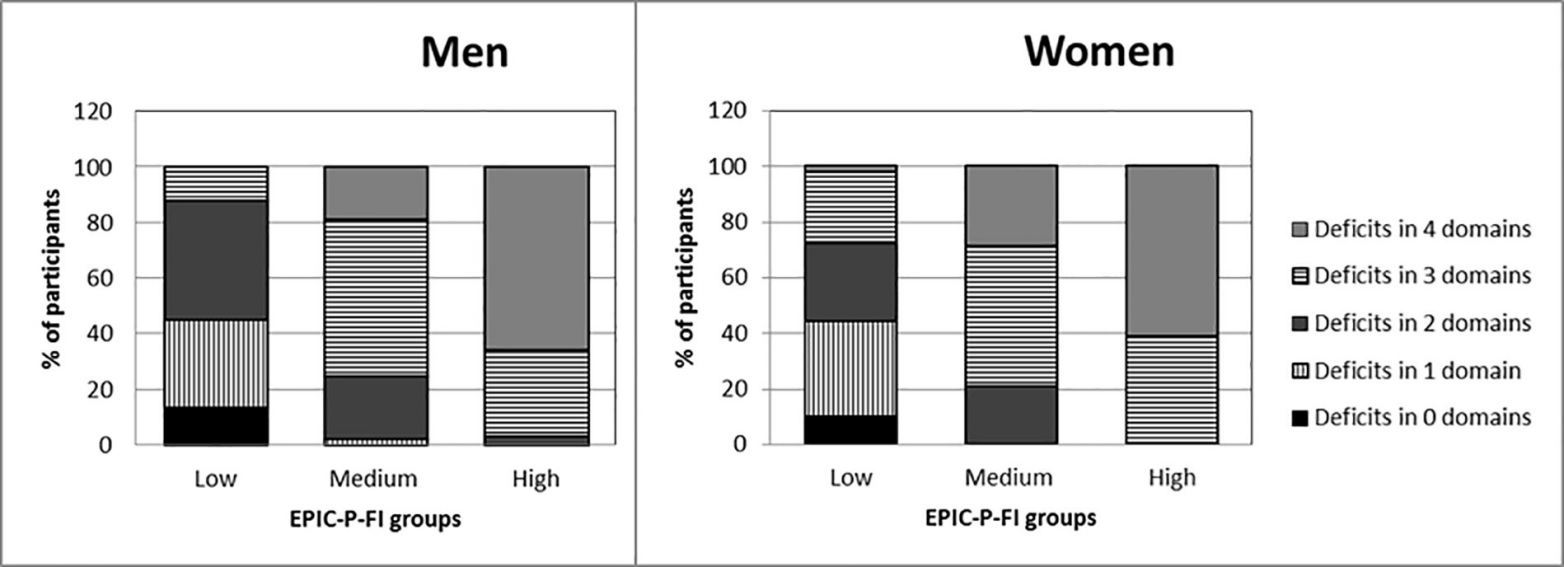
Number of domains with deficits in the low, medium and high EPIC-P-FI group. Stacked bar graphs show the percentage of participants with deficits in none (black), 1 (vertical stripes), 2 (dark grey), 3 (horizontal stripes) or 4 (light grey) of the domains for men (left) and women (right) in the low, medium and high EPIC-P-FI group based on EPIC-P-FI score tertiles.

The lowest correlation was between the EPIC-P-FI score and physiological aspects in men and women (ρ = 0.51, P<0.001 and ρ = 0.52, P<0.001 respectively). Of the four domains, the health domain presented the highest correlation with age of men and women (ρ = 0.36, P<0.001 and ρ = 0.30, P<0.001 respectively). The correlation between individual domains ranged from 0.05 (P = 0.2) to 0.54 (P<0.001). Most people in the high EPIC-P-FI group had deficits in three or four domains, with more than 50% of people having deficits in all four domains (Fig 3). In contrast, in the low EPIC-P-FI group most people had no deficits or deficits in only one or two domains.

### Distribution of characteristics among the EPIC-Potsdam study population across categories of frailty

Characteristics of the EPIC-Potsdam study population are presented in Tables 2 and 3. A total of 410 men and 402 women were included in the analyses. The mean age was 67 years for men and 65 years for women at the time of examination. Participants with missing data for handgrip strength were older (men 70 years vs 67 years P = 0.004; women 67 years vs 65 years P = 0.001) and had higher EPIC-P-FI values (men 0.23 vs 0.15, P<0.001; women 0.22 vs 0.17, P = 0.04) than participants with available hand grip strengths measurements. Participants with missing data for PAL were not different to participants with available PAL measurements. Sensitivity analysis indicated that the reduced FI did not significantly differ from the complete EPIC-P-FI.

**Table 2:** Characteristics of 410 Men in the EPIC-Potsdam Sub-Study Population by EPIC-P-FI Score in 2010.

		Men				
		EPIC-P-FI groups				
	Variable	Low	Medium	High	*P*-trend	Total
	**FI score (mean (min—max))**	0.04 (0.00–0.08)	0.14 (0.09–0.20)	0.31 (0.21–0.60)		0.16 (0.00–0.60)
**Socio-**	**Age in years (mean (SE))**	64.3 (0.7)	66.7 (0.6)	69.6 (0.7)	<0.001	66.9 (0.4)
**demographic**	**Living with other people (%)**^**a**^	95.1	90.6	79.9	<0.001	88.5
**factors**	**Having a partner (%)**^**a**^	93.3	87.9	79.4	0.001	86.8
	**Profession (%)**^**a**^ ^**b**^					
	**higher grade professional**	45.1	34.3	25.0		34.6
	**lower grade professional**	37.4	45.0	54.2		45.6
	**skilled/non-manual worker**	17.1	16.0	15.2		16.1
	**simple manual worker**	0.4	4.7	5.7		3.7
	**Education (%)**^**a**^					
	**no vocational/vocational training**	18.6	31.4	38.6		29.8
	**technical college**	12.5	14.9	19.4		15.6
	**university**	68.9	53.7	41.9		54.6
**Lifestyle**	**Alcohol g/day (mean (SE))**^**a**^	14.6 (1.6)	16.5 (1.5)	16.6 (1.6)	0.378	16.0 (0.9)
**factors**	**Smoking status (%)**^**a**^					
	**never smoker**	45.5	34.5	17.9		32.6
	**ex-smoker**	46.3	57.4	65.6		56.5
	**Smoker**	8.3	8.0	16.5		10.9
	**Sport h/week (mean (SE))**^**a**^	3.0 (0.3)	2.2 (0.3)	2.2 (0.3)	0.067	2.5 (0.2)
	**Walking h/week (mean (SE))**^**a**^	7.0 (0.7)	7.8 (0.6)	8.9 (0.7)	0.058	7.9 (0.4)
	**DIY h/week (mean (SE))**^**a**^	5.1 (0.6)	4.0 (0.5)	4.7 (0.6)	0.602	4.6 (0.3)
	**Housework h/week (mean (SE))**^**a**^	4.8 (0.6)	5.2 (0.6)	6.2 (0.6)	0.104	5.4 (0.3)
	**Cycling h/week (mean (SE))**^**a**^	3.7 (0.3)	3.1 (0.3)	2.4 (0.3)	0.002	3.1 (0.2)
	**Gardening h/week (mean (SE))**^**a**^	5.1 (0.5)	5.2 (0.5)	5.3 (0.5)	0.769	5.2 (0.3)
	**Sleep day h/day (mean (SE))**^**a**^	0.4 (0.08)	0.6 (0.07)	0.7 (0.08)	0.018	0.6 (0.04)
	**Sleep night h/night (mean (SE))**^**a**^	7.3 (0.08)	7.4 (0.08)	7.3 (0.09)	0.990	7.3 (0.05)
	**Watch TV h/day (mean (SE))**^**a**^	2.7 (0.1)	2.9 (0.1)	3.3 (0.1)	0.003	3.0 (0.1)
**Biological**	**CRP in μg/l (mean (SE))**^**a**^	1.8 (0.6)	1.8 (0.5)	4.3 (0.6)	0.002	2.6 (0.3)
**factors**	**Blood pressure in mm Hg (mean (SE))**^**a**^					
	**Diastolic**	79.7 (0.8)	79.8 (0.7)	78.6 (0.8)	0.341	79.4 (0.5)
	**Systolic**	137.3 (1.3)	138.6 (1.2)	136.4 (1.3)	0.651	137.5 (0.7)
	**VO**_**2**_**max (mean (SE))**^**a**^	29.8 (0.5)	28.9 (0.5)	29.3 (0.6)	0.504	29.3 (0.3)

^a^ values are age-adjusted

^b^ current profession or if retired, the last carried out profession

**Table 3:** Characteristics of 402 Women in the EPIC-Potsdam Sub-Study Population by EPIC-P-FI Score in 2010.

		Women				
		EPIC-P-FI score				
	Variable	Low	Medium	High	*P*-trend	Total
	**FI score (mean (min—max))**	0.05 (0.00–0.10)	0.16 (0.11–0.22)	0.33 (0.22–0.70)		0.18 (0–0.7)
**Socio-**	**Age in years (mean (SE))**	63.2 (0.7)	65.0 (0.7)	66.9 (0.8)	<0.0001	65.0 (0.4)
**demographic**	**Living with other people (%)**^**a**^	75.7	77.3	71.6	0.443	74.9
**factors**	**Having a partner (%)**^**a**^	73.3	75.9	70.2	0.574	73.1
	**Profession (%)**^**a**^^,^ ^**b**^				
	**higher grade professional**	16.8	21.2	25.0		21.0
	**lower grade professional**	70.3	68.6	60.5		66.5
	**skilled/non-manual worker**	12.0	8.0	11.6		10.5
	**simple manual worker**	0.9	2.2	2.9		2.0
	**Education (%)**^**a**^				
	**no vocational/vocational training**	33.5	32.1	41.5		35.7
	**technical college**	32.2	32.9	26.9		30.7
	**university**	34.3	35.0	31.6		33.7
**Lifestyle**	**Alcohol g/day (mean (SE))**^**a**^	10.7 (1.0)	10.6 (1.0)	7.0 (1.0)	0.013	9.4 (0.06)
**factors**	**Smoking status(%)**^**a**^				
	**never smoker**	61.1	58.5	62.2		60.6
	**ex-smoker**	27.0	32.0	29.1		29.4
	**Smoker**	11.8	9.4	8.7		10.0
	**Sport h/week (mean (SE))**^**a**^	2.8 (0.3)	2.2 (0.3)	2.5 (0.3)	0.530	2.5 (0.2)
	**Walking h/week (mean (SE))**^**a**^	7.4 (0.5)	6.9 (0.5)	7.1 (0.5)	0.694	7.2 (0.3)
	**DIY h/week (mean (SE))**^**a**^	1.3 (0.3)	1.2 (0.3)	0.6 (0.3)	0.203	1.0 (0.2)
	**Housework h/week (mean (SE))**^**a**^	14.3 (0.7)	13.5 (0.7)	14.3 (0.7)	0.980	14.0 (0.5)
	**Cycling h/week (mean (SE))**^**a**^	3.5 (0.3)	2.6 (0.3)	2.3 (0.3)	0.013	2.8 (0.2)
	**Gardening h/week (mean (SE))**^**a**^	4.5 (0.4)	4.2 (0.4)	4.3 (0.4)	0.742	4.3 (0.3)
	**Sleep day h/day (mean (SE))**^**a**^	0.2 (0.04)	0.2 (0.04)	0.4 (0.04)	0.002	0.3 (0.03)
	**Sleep night h/night (mean (SE))**^**a**^	7.4 (0.1)	7.1 (0.1)	7.0 (0.1)	0.011	7.2 (0.1)
	**Watch TV h/day (mean (SE))**^**a**^	2.8 (0.1)	2.8 (0.1)	3.0 (0.1)	0.178	2.9 (0.07)
**Biological**	**CRP in μg/l (mean (SE))** ^**a**^	2.5 (0.6)	3.2 (0.5)	4.4 (0.6)	0.019	3.4 (0.3)
**factors**	**Blood pressure in mm Hg (mean (SE))** ^**a**^					
	**diastolic**	77.4 (0.8)	79.7 (0.8)	78.3 (0.8)	0.437	78.5 (0.5)
	**systolic**	133.3 (1.4)	136.3 (1.4)	133.0 (1.4)	0.914	134.2 (0.9)
** **	**VO**_**2**_**max (mean (SE))**^**a**^	28.0 (0.4)	26.8 (0.4)	26.8 (0.5)	0.045	27.3 (0.3)

^a^ values are age-adjusted

^b^ current profession or if retired, the last carried out profession

A small percentage of participants were current smokers (11% of men and 10% of women). Many of the participants stopped smoking (57% of men and 29% of women). The minority of men (12%) and women (25%) were single and lived on their own. Around one third of the participants (37% for men and women) were still working.

Men with high EPIC-P-FI scores were older, more likely to be without a partner and live alone, less educated and less likely to work (Table 2). In women, the same trends could be observed (Table 3). Since the groups of men and women with a high FI were older, the mean values for lifestyle and biological factors were age-adjusted. Men with high EPIC-P-FI values were more likely to smoke, sleep more during the day, cycle less and watch more TV than men with low EPIC-P-FI values (Table 2). Women with high EPIC-P-FI values were more likely to consume less alcohol, watch more TV, sleep more during the day and less during the night and cycle less than women with low EPIC-P-FI scores (Table 3).

Regarding biological factors, both men and women with high EPIC-P-FI values had higher C-reactive protein (CRP) levels than participants with a low EPIC-P-FI score (Tables 2 and 3). In women, VO2max levels were negatively associated with the EPIC-P-FI values. In men, no meaningful associations with the EPIC-P-FI values were observed with blood pressure or cardio-metabolic fitness measured by VO2max. Most of the individuals in the high EPIC-P-FI group had deficits in three or four domains, with more than 50% of people having deficits in all four domains. In contrast, in the low EPIC-P-FI group most people had no deficits or deficits in only one or two domains (Fig 3).

## Discussion

The current study provides a multidimensional tool to measure healthy ageing and frailty. This tool was established following an already published guideline and based on health-related questions and examinations conducted in a US cohort of older adults [11]. This newly developed EPIC-P-FI included 32 deficit variables. Men appeared to be slightly less affected by frailty compared to women, with frailty increasing with age in both groups. The physical ability domain showed the highest correlation with the total EPIC-P-FI and the lowest with the physiological domain. Individuals with a high FI value were more likely to sleep more during the day and less during the night, were less physical active i.e. cycling, watched more TV and had higher CRP levels than those with low FI values.

Frailty represents a state of heightened vulnerability in the aging process [25]. In the clinical setting, the Fried’s phenotype scale with its five evaluations is a convenient tool and satisfies the applicants through its quick assessment. However, in epidemiological studies, the measurement of frailty through a FI seems to be more useful [26]. By concept, the accumulation of deficit approach allows the assessment of overall frailty, but also specific deficits and domains and thus, is more suitable to provide a mechanistic understanding of the aging phenomenon [27]. The FI is a robust tool independently of the number of deficits used per domain and also of the number of domains [28–30]. The study by Abete et al showed [31] FI as valid measure of frailty after comprehensive geriatric assessment in an Italian cohort of non-institutionalized patients. Further use of this FI in Italian outpatients older than 65 indicated higher comorbidity and disability in patients with chronic heart failure (CHF), where increased more in presence than in the absence of CHF, with increasing frailty [32]. In the current study, the deficits had been grouped into four domains (health, physical ability, psychosocial and physiological domains). Most of the study participants with highly EPIC-P-FI values had deficits in three or four domains. Deficits were seen in all possible combinations of domains which indicate that frailty presents itself differently in each individual. It is still unclear which domains and which combinations thereof are the most important contributors or indicators of frailty and its health consequences and if it is possible for individuals to compensate deficits in one domain with resources in another domain [33]. Although the multidimensional approach (using nutritional status, physical activity, cognition, mood, social relations and psychological health among others) to measure frailty has been frequently used, only few of the studies reported the impact of individual domains [34–42]. Our study supported the view that several domains should be used for the assessment of frailty and should also be analyzed individually [36, 41]. We should take into account that accuracy and precision can be for selected domains can be differentiated (for example higher for health domain)

The EPIC-P-FI characteristics, such as range, distribution and association with age in the EPIC-Potsdam cohort are similar to those of other published FIs [3, 28, 43–52]. The approach of calibrating the obtained points to the total number of variables is very straightforward, particularly in respect to comparability with other study results. In our experience, neither the number of variables, nor a difference in estimating a deficit will affect the comparability of different FIs if the same principle of constructing the FI had been used. Further, although not a validation in itself, the characteristics indicate that the application of the EPIC-P-FI can generate meaningful results. Sensitivity of the FI was evaluated with a reduced FI calculated with 23 deficits (excluding prevalent chronic diseases). This FI was compared with the complete EPIC-P-FI. This analysis could be considered as a proxy analysis compared to a full analysis of the validity. Especially the increase of the FI with age is considered an indicator of construct validity [3], even though it is just consequential that the total FI score also increases with age if each deficit increases with age [11].

Although frailty differs in men and women, many studies did not analyze frailty separately for gender [42, 46, 53–56]. It is still unknown why women acquire more deficits at a given age but have a lower mortality than men [57]. There may be some factors affecting life expectancy in older people that are not captured by current frailty measures and that these factors are present more often in men than in women. To account for and learn more about gender differences, men and women were analyzed separately in the current study. Having no partner and living alone was associated with frailty in men but not in women while lower education was associated with frailty in men and women, as previously shown [35, 58–63].

Those who were frail were less likely to be active socially. On the other hand, it is possible that active participation in society may retard the onset of frailty [62]. An association between smoking and frailty was observed in men, but not in women in the EPIC-Potsdam cohort. Most studies showed a positive association between smoking and frailty in both genders [56]. Similar to our results, Wang et al. found that smoking and frailty were only associated in men and not in women [55]. As suggested by Woo et al [62] this may be a survivor effect, in that those who were susceptible to smoking-related diseases might have died at an earlier age, and an elderly cohort population is largely consisting of such survivors.

Consuming moderate amounts of alcohol was negatively associated with frailty in women but not in men in the EPIC-Potsdam cohort. Only a few studies have previously analyzed the impact of drinking alcohol on frailty and found that moderate alcohol intake was associated with less frailty in both genders [62, 63]. There was also a trend that frail people slept more during the day and less at night. Mixed results have been published about sleep duration and frailty [64–65]. One reason for this might be the U-shaped association between sleep duration and health. It has been shown that frailty and a long night time sleep duration of 10 or more hours were independently associated with 5-year mortality in older adults [64].

Watching TV was positively associated with frailty in men and women in the EPIC-Potsdam cohort, which is in line with the known link between sedentary behavior and unfavorable health outcome [66–68]. The mechanisms through which television time is associated with metabolic risk, even in this healthy subpopulation, are likely to be of both physiological and behavioral origin. Physiologically, there is emerging evidence that sedentary behavior results in unique metabolic outcomes. Behaviorally, it has been suggested that sedentary time is associated with health outcomes as it displaces time in only a single leisure-time sedentary behavior was measured [66]. On the other hand, no association was observed between self-reported physical activity (except cycling) and frailty. This might be explained by known discrepancies between self-reported and objectively measured physical activity [69]. An association was seen between the physical fitness measured by VO2max and frailty in women, but not in men in the EPIC-Potsdam cohort.

Chronic inflammation has been shown to be associated with frailty and CRP levels have often been used as a marker for inflammation [70, 71]. It was expected that CRP will be more strongly predictive of frailty incidence in women, but accordingly, men and women with high EPIC-P-FI values had higher levels of CRP than people with low EPIC-P-FI values. However, the underlying pathomechanisms are still poorly defined. CRP levels negatively correlated with the rate of skeletal muscle protein synthesis, which may support the idea that low-grade inflammation is implicated in sarcopenia development in frail people [71].

### Limitations and strengths

Selection bias might be more pronounced in elderly people, and especially frail people might be underrepresented since they refuse to endure long procedure of examinations. Another problem in generating a FI is the choice of variables and domains and the categorization there, since there is no gold standard yet. So far, the EPIC-P-FI must be considered to be in a developmental stage and therefore, should be validated before applying it to other populations. One of the strengths is the use of the population-based prospective EPIC-Potsdam study whose participants underwent a highly standardized procedure of examinations. The multidimensional EPIC-P-FI developed for this study covers four important domains instead of just the physical domain [7]. The combination of objectively measured and self-reported data is a strong point of this study as is the inclusion of middle-aged people (50–65 years) who are often excluded from frailty research [72] but may give the opportunity to investigate the early changes leading to frailty.

## Conclusions

The multidimensional EPIC-P-FI produced a rich assessment of frailty in the EPIC cohort. It is of high importance to investigate frailty separately in both genders since different factors might be involved in the development of frailty in men and women. The EPIC cohort, monitored for more than 20 years, offers the potential to analyze lifestyle factors, health transitions, mortality and frailty prospectively and retrospectively. Exploring frailty in this well-established German population will provide further insights on ageing-associated processes, help to identify factors that predispose to frailty and thereby promote healthy longevity.

## Supporting information

S1 Table Prevalence of EPIC-FI Deficits in 410 Men of the EPIC-Potsdam Sub-Study Population in 2010. (PDF)

S2 Table Prevalence of EPIC-FI Deficits in Women of the EPIC-Potsdam Sub-Study Population in 2010. (PDF)
