# Supplementary material for: The accumulation of deficits approach to describe frailty
Source: PLoS One. 2019 Oct 15;14(10):e0223449. doi: 10.1371/journal.pone.0223449 (PMC6793873; doi:10.1371/journal.pone.0223449)
Supplement: S1 Table — (PDF) [file pone.0223449.s001.pdf]

Supporting information

S1 Table Prevalence of EPIC-P-FI Deficits in 410 Men of the EPIC-Potsdam Sub-Study Population in 2010

	Men				
**Domain**	**Description**	**EPIC-P-FI score**			**Total**
		**Low N = 128**	**Medium N = 149**	**High N = 133**	**N = 410**
**Health**	**Pain (%)**	19.5	51.0[aa]	82.7[aa]	51.5
	**State of health (%)**				
	**0.5 (less well)**	0.0	8.7[aa]	45.1[aa]	17.8
	**1 (bad)**	0.0	0.0	1.5	0.5
	**More than 5 medications (%)**	6.3	15.4[a]	44.4[aa]	22.0
	**Tumor (%)**	1.6	7.5[a]	21.1[aa]	10.1
	**Missing subjects (N)**	4	2	0	6
	**Diabetes (%)**	1.6	8.9[aa]	21.8[aa]	10.8
	**Missing subjects (N)**	1	3	0	4
	**Myocardial infarction (%)**	2.3	2.7	7.5	4.1
	**Stroke (%)[b]**	0	2.0	6.8[aa]	2.9
	**Transient Ischemic Attack (TIA) (%)[b]**	2.3	1.3	6.0	3.2
	**Heart failure (%)[b]**	0	0.7	5.3[a]	2.0
	**Angina pectoris (%)**	0.8	8.5[aa]	10.5[aa]	6.6
	**Missing subjects (N)**	1	7	9	17
	**Hypertension (%)**	32.8	61.3[aa]	81.4[aa]	59.0
	**Missing subjects (N)**	6	7	4	17
	**Osteoporosis (%)[b]**	0	1.4	3.2	1.5
	**Missing subjects (N)**	0	2	8	10
**Psychosocial Aspects**	**Managed less than wanted because of mental problems (%)**	1.6	10.7[aa]	34.6[aa]	15.6
	**Working carefully as usual not possible (%)**	0.8	6.0[a]	28.6[aa]	11.7
	**Satisfied with health (%)**				
	**0.5 (rather dissatisfied)**	3.1	5.4	38.3[aa]	15.4
	**1 (very dissatisfied)**	0.0	0.0	1.5	0.5
	**Satisfied with life (%)**				
	**0.5 (rather dissatisfied)**	1.6	3.4	24.8[aa]	9.8
	**1 (very dissatisfied)**	0	0	0	0
	**Dispirited and sad (%)[b]**				
	**0.5 (sometimes, quite often)**	0	2.0[a]	11.3[aa]	4.4
	**1(mostly, always)**	2.3	0	2.3	1.5
	**Have still many plans (%)**				
	**0.5 (partially applies)**	36.2	38.4	48.8	41.0
	**1 (does not apply)**	3.9	9.6	16.5	10.0
	**Missing subjects (N)**	1	3	6	10
	**Affected contacts to other persons (%)**				
	**0.5 (sometimes)**	0.8	6.7[a]	19.5[aa]	9.0
	**1 (always, mostly)**	0	0	6.8	2.2
**Physical Ability**	**Managed less than I wanted because of physical problems(%)**	1.6	18.1[aa]	60.2[aa]	26.6
	**Only done specific things (%)**	0	12.1[aa]	57.1[aa]	22.9
	**Hand grip strength (%)**	1.7	7.1[a]	25.7[aa]	10.8
	**Missing subjects (N)**	8	8	24	40
	**Physical activity level (%)**	7.0	13.4	42.6[aa]	21.0
	**Missing subjects (N)**	14	30	18	62
	**Limited in medium heavy activities (%)[b]**				
	**0.5 (minor limitations)**	2.3	22.8[aa]	59.4[aa]	28.3

	**1 (major limitations)**	0	0.7	9.8	3.4
	**Limited because of pain (%)**				
	**0.5 (minor limitations)**	18.8	47.0	70.7[aa]	45.9
	**1 (major limitations)**	0	2.7	16.5	6.3
	**Limited in climbing stairs (%)[b]**				
	**0.5 (minor limitations)**	3.9	27.5[aa]	59.4[aa]	30.5
	**1 (major limitations)**	0	1.3	9.0	3.4
	**Full of energy (%)[b]**				
	**0.5 (rarely)**	0	1.3	14.3[aa]	5.1
	**1 (never)**	0	0.7	3.0	1.2
**Physiological Aspects**	**Body fat percentage (%)**	1.6	23.0[aa]	33.3[aa]	19.6
	**Unwanted weight loss (%)**	2.5	7.0	9.9[a]	6.4
	**Missing subjects (N)**	8	7	22	37
	**BMI (%)**	0.8	27.5[aa]	41.4[aa]	23.7
	**Whrt (%)**	6.3	43.6[aa]	63.9[aa]	38.5
	**Malnutrition (%)**	9.4	22.8[aa]	32.3[aa]	21.7

[a] *P*-value < 0.05 compared to low EPIC-P-FI; [aa]*P*-value <0.01 compared to low EPIC-P-FI; [b]Differences between EPIC-P-FI groups were calculated with Fisher's exact test.
