# Supplementary material for: The accumulation of deficits approach to describe frailty
Source: PLoS One. 2019 Oct 15;14(10):e0223449. doi: 10.1371/journal.pone.0223449 (PMC6793873; doi:10.1371/journal.pone.0223449)
Supplement: S2 Table — (PDF) [file pone.0223449.s002.pdf]

Supporting information

S2 Table Prevalence of EPIC-P-FI Deficits in Women of the EPIC-Potsdam Sub-Study Population in 2010

	Women				
**Domain**	**Description**	**EPIC-P-FI score**			**Total**
		**Low N = 133**	**Medium N = 137**	**High N = 132**	**N = 402**
**Health**	**Pain (%)**	35.3	70.8[aa]	90.2[aa]	65.4
	**State of health (%)**				
	**0.5 (less well)**	2.3	9.5[a]	52.3[aa]	21.1
	**1 (bad)**	0	0	3.0	1.0
	**More than 5 drugs (%)**	3.0	13.9[aa]	40.2[aa]	18.9
	**Tumor (%)**	4.5	11.9[a]	14.6[aa]	10.4
	**Missing subjects (N)**	1	3	2	6
	**Diabetes (%)**	1.5	4.4	7.8[a]	4.5
	**Missing subjects (N)**	0	2	4	6
	**Myocardial infarction (%)[b]**	0	0	3.0	1.0
	**Stroke (%)[b]**	0	1.5	2.3	1.2
	**Tia (%)[b]**	2.3	3.6	9.1[a]	5.0
	**Heart failure (%)[b]**	0	1.5	0	0.5
	**Missing subjects (N)**	0	0	1	1
	**Angina pectoris (%)[b]**	1.5	2.3	6.5	3.3
	**Missing subjects (N)**	1	4	8	13
	**Hypertension (%)**	27.6	45.9[aa]	70.5[aa]	47.7
	**Missing subjects (N)**	6	2	10	18
	**Osteoporosis (%)**	3.1	6.3	8.3	5.8
	**Missing subjects (N)**	4	9	12	25
**Psychosocial Aspects**	**Managed less than wanted because of mental problems (%)**	3.0	15.3[aa]	49.2[aa]	22.4
	**Working carefully as usual not possible (%)**	2.3	8.8[a]	43.9[aa]	18.2
	**Satisfied with health (%)**				
	**0.5 (rather dissatisfied)**	0	9.5[aa]	45.5[aa]	18.2
	**1 (very dissatisfied)**	0	0	5.3	1.7
	**Satisfied with life (%)**				
	**0.5 (rather dissatisfied)**	0	5.1[aa]	17.4[aa]	7.5
	**1 (very dissatisfied)**	0	0	3.8	1.2
	**Dispirited and sad (%)[b]**				
	**0.5 (sometimes, quite often)**	3.0	4.4	18.2[aa]	8.5
	**1(mostly, always)**	0	2.9	6.1	3.0
	**Have still many plans (%)**				
	**0.5 (partially applies)**	38.2	43.0	46.1[aa]	42.4
	**1 (does not apply)**	3.1	6.7	16.4	8.6
	**Missing subjects (N)**	2	2	4	8
	**Affected contacts to other persons (%)**				
	**0.5 (sometimes)**	3.8	10.9[a]	29.5[aa]	14.7
	**1 (always, mostly)**	0	0	13.6	4.5
**Physical Ability**	**Managed less than wanted because of physical problems (%)**	3.8	27.7[aa]	77.3[aa]	36.1
	**Only done specific things (%)**	0.8	17.5[aa]	71.2[aa]	29.6
	**Hand grip strength (%)[b]**	1.6	5.0	15.1[aa]	6.8
	**Missing subjects (N)**	8	17	26	51
	**Physical activity level (%)**	6.3	19.8[aa]	31.5[aa]	19.1
	**Missing subjects (N)**	22	21	24	67
	**Limited in medium heavy activities (%)**				

		**0.5 (minor limitations)**	6.0	41.6[aa]	61.4[aa]	36.3
		**1 (major limitations)**	0	0.7	15.9	5.5
	**Limited because of pain (%)**					
		**0.5 (minor limitations)**	26.3	70.1[aa]	70.5[aa]	55.7
		**1 (major limitations)**	1.5	2.9	24.2	9.5
	**Limited in climbing stairs (%)**					
		**0.5 (minor limitations)**	7.5	46.0[aa]	66.7[aa]	40.0
		**1 (major limitations)**	0	2.2	15.9	6.0
	**Full of energy (%)[b]**					
		**0.5 (rarely)**	1.5	2.9	24.3[aa]	9.5
		**1 (never)**	0	0.7	6.1	2.2
**Physiological Aspects**	**Body fat percentage (%)**		5.3	21.9[aa]	32.1[aa]	19.7
	**Unwanted weight loss (%)**		4.8	7.4	4.5	5.6
		**Missing subjects (N)**	8	16	22	46
	**BMI (%)**		0.8	23.4[aa]	50.0[aa]	24.6
	**Whrt (%)**		2.3	31.4[aa]	53.8[aa]	29.1
	**Malnutrition (%)**		15.0	31.4[aa]	45.5[aa]	30.6

[a] *P*-value < 0.05 compared to low EPIC-P-FI; [aa]*P*-value <0.01 compared to low EPIC-P-FI; [b]Differences between EPIC-P-FI groups were calculated with Fisher's exact test.
